# DMNQ induces ferroptosis and augments the efficacy of anti-PD-L1 immunotherapy in gastric cancer via the STAT3/SLC1A4 axis to mediate cysteine metabolism reprogramming

**DOI:** 10.1016/j.redox.2026.104055

**Published:** 2026-01-26

**Authors:** Wenshuai Zhu, He Qi, Fubo Jing, Yuxuan Shi, Yuanxin Xing, Xiaoli Ma, Bin Ning, Yunshan Wang, Yanfei Jia

**Affiliations:** a Research Center of Basic Medicine, Central Hospital Affiliated to Shandong First Medical University, Jinan, 250013, People's Republic of China; b Department of Medical Laboratory, Shandong Second Medical University, Weifang, 261053, People's Republic of China; c Research Center of Basic Medicine, Jinan Central Hospital, Shandong University, Jinan, 250013, People's Republic of China; d Central Hospital Affiliated to Shandong First Medical University, Shandong First Medical University, Jinan, 250013, People's Republic of China

**Keywords:** DMNQ, Ferroptosis, STAT3/SLC1A4 axis, Cysteine metabolism, Anti-PD-L1 immunotherapy

## Abstract

Ferroptosis plays an essential role in tumor progression. Therapeutic agents targeting ferroptosis emerge as a novel strategy for cancer treatment. Abnormal amino acid metabolism can control ferroptosis sensitivity in cancer cells, and lead to the deficiency or accumulation of specific products in the tumor microenvironment (TME). Here, we demonstrated that 2,3-dimethoxy-1,4-naphthoquinone (DMNQ) induced growth inhibition in gastric cancer cell lines, primary gastric cancer mouse models, and patient-derived tumor organoids. DMNQ exerted ferroptosis inducing effects by inhibiting STAT3 phosphorylation and transcriptional activity. Importantly, the STAT3/SLC1A4 axis regulated cysteine uptake, tumor killing by T cells and the efficacy of anti-PD-L1 immunotherapy. Collectively, our findings revealed a critical mechanism by which DMNQ exerts a significant anti-cancer role in gastric cancer through increasing ferroptosis to enhance cancer immunotherapy and may provide a novel therapeutic strategy for gastric cancer.

## Highlights

• DMNQ induces ferroptosis in gastric cancer cells by targeting the SH2 domain of STAT3 and inhibiting its transcriptional activity.
• DMNQ reprograms cysteine metabolism through STAT3/SLC1A4 axis.
• STAT3/SLC1A4 axis regulates ferroptosis, tumor killing by T cells and the efficacy of anti-PD-L1 immunotherapy in gastric cancer.
• DMNQ boosts anti-tumor effects with anti-PD-L1 immunotherapy.

## 1 Introduction

Gastric cancer ranks as the fourth leading cause of cancer-related death worldwide. Owing to the limitations of early screening technologies, most gastric cancer patients present with advanced-stage disease at diagnosis and require systemic therapies [1,2]. Chemotherapy, radiotherapy, and immune checkpoint inhibitors (ICIs) are the preferred treatments for unresectable and advanced gastric cancer [[3], [4], [5]]. However, the majority of patients do not benefit from those treatments because of resistance and "immunologically cold" tumors, which are characterized by a tumor microenvironment (TME) [3,6]. Therefore, the identification of innovative pharmacological agents is imperative for improving the outcomes of patients with gastric cancer.

Increasing evidence has highlighted the critical role of ferroptosis in cancer therapies [7]. The core mechanism of ferroptosis involves iron accumulation leading to the generation of intracellular reactive oxygen species (ROS), which subsequently trigger lipid peroxidation and ultimately result in cell death. ROS and the metabolic basis of ferroptosis in cancer cues impact both cancer cell and immune populations [[8], [9], [10], [11]]. Recently, drugs that target ferroptosis have been shown to sensitize ICIs [12,13]. To identify candidates with ferroptosis-inducing activities in gastric cancer, we screened small-molecule compounds from a commercial chemical library previously reported to modulate ROS levels. We identified 2,3-dimethoxy-1,4-naphthoquinone (DMNQ), which has the greatest inhibitory effect on gastric cancer cells and whose anti-cancer effect in gastric cancer treatment has not yet been investigated. While earlier studies have shown that DMNQ broadly induces ROS production and apoptosis in tumors [14], its relationship with ferroptosis and immunotherapy in cancer remains unexplored. Our study demonstrated that DMNQ induces ROS accumulation in gastric cancer cells by targeting the Src homology 2 (SH2) domain of STAT3, ultimately leading to ferroptosis.

Tumor cells are compelled to undergo metabolic reprogramming to adapt to hypoxia, stress, and unrestricted proliferation conditions [15]. Abnormal amino acid metabolism, such as glutamic acid, glutamine, serine, and cysteine metabolism, can control ferroptosis sensitivity in cancer cells, and lead to the deficiency or accumulation of specific products in the TME [[16], [17], [18], [19], [20]]. As cysteine is often a limiting nutrient in the TME, ensuring its supply to sustain cancer cell survival is critical. Cellular cysteine is an indispensable substrate for the synthesis of GSH and its uptake relies on solute carrier (SLC) transporters [21]. SLC1A4 is a sodium-dependent neutral amino acid transporter for cysteine, alanine, serine, and threonine [22]. SLC1A4 transports serine and has been implicated in the progression of GC [23]. Recent studies have shown that cysteine supports tumor resistance to ferroptosis [20,21,24]. Nevertheless, it remains unclear whether SLC1A4 contributes to cysteine uptake in cancer cells, consequently regulating ferroptosis.

STAT3, a pivotal signaling molecule in cellular signal transduction, can indirectly reshape the tumor metabolic landscape by influencing other key signaling pathways [25,26]. However, the mechanism by which STAT3 regulates amino acid metabolism in gastric cancer remains unclear. In the present study, by employing diverse mouse models, organoids, and gastric cancer cells, we demonstrated that DMNQ exerted ferroptosis-inducing effects by inhibiting STAT3 activation. STAT3 can directly bind to the promoter regions of the cysteine transporter SLC1A4. The STAT3/SLC1A4 axis regulated cysteine uptake, tumor killing by T cells and the efficacy of anti-PD-L1 (αPD-L1) immunotherapy. Overall, our study provides a new therapeutic strategy to combine ferroptosis and immunotherapy for gastric cancer treatment.

## 2 Materials and methods

### 2.1 Cell culture and reagents

Gastric cancer (GC) cell lines (MKN-45 and MFC) were obtained from the Shanghai Institute of Biochemistry and Cell Biology, Chinese Academy of Sciences (Shanghai, China). These cell lines were maintained in RPMI 1640 medium (MACGENE; HyClone, USA) supplemented with 10 % fetal bovine serum (FBS). The cells were cultured in a humidified incubator with 5 % CO_2_ at 37 °C (Thermo Fisher Scientific, MA, USA). All the cell lines were routinely tested for mycoplasma contamination to ensure that there was no microbial interference. Additionally, short tandem repeat (STR) profiling was performed for all the cell lines to confirm their identity and avoid cross-contamination.

### 2.2 Chemicals and reagents

Antibodies specific for phospho-STAT3-Tyr705 (AP0070; 1:2000 for WB, 1:200 for immunofluorescence, 1:500 for immunohistochemistry), STAT3 (A19566; 1:2000 for WB), SLC1A4 (A12507; 1:1000 for WB), 4-HNE (A24456; 1:200 for immunohistochemistry), and CD8A (A0663; 1:200 for immunofluorescence, 1:500 for immunohistochemistry) were purchased from ABclonal (Wuhan, China). Antibodies specific for Ki67 (27309-1-AP; 1:2000 for immunohistochemistry), GZMB (13588-1-AP; 1:200 for immunofluorescence), and GAPDH (60004-1-Ig; 1:10000 for WB) were purchased from Proteintech (Wuhan, China).

### 2.3 Cell transfection and lentivirus infection

The STAT3 and SLC1A4 small interfering RNA (siRNA) or respective negative control (NC) siRNA were purchased from Obio Technology (Shanghai, China). The siRNAs were transfected into cells utilizing Opti-MEM and Lipofectamine 2000 transfection reagent (Invitrogen, CA, USA) at a concentration of 100 nM following the manufacturer's guidelines. The STAT3-overexpressing lentivirus (STAT3) and empty vector (EV) were constructed by GENECHEM (Shanghai, China). Thecorresponding sequence information is listed in Table S1.

### 2.4 Tumor organoid culture and transfection

Fresh clinical tumor tissues were minced with sterile scissors and forceps under aseptic conditions and then digested with tumor tissue digestion solution (Mogengel Bio, Xiamen, China). Digestion was terminated by adding FBS, and the mixture was centrifuged to collect the tissue pellet, which was washed twice with DPBS. The pellet was resuspended in Matrigel (Mogengel Bio) and added to 24-well plates. After the Matrigel solidified, Gastric Cancer Organoid Complete Medium (Mogengel Bio) was added, and the plates were cultured in a humidified 5 % CO_2_ incubator at 37 °C.

For 24-well plates with precultured gastric cancer organoids, Matrigel was added, and the mixture was incubated for solidification. The transfection mixture was prepared with Gastric Cancer Organoid Complete Medium (supplemented with 10 % FBS), siRNA (100 nM final concentration), STAT3 plasmid (3 μg final mass per well), and Lipofectamine 2000 Transfection Reagent (Invitrogen, USA). The mixture was incubated at room temperature to form nucleic acid‒liposome complexes. The prewarmed mixture was added to the wells, incubated at 37 °C in a 5 % CO_2_ incubator, and then replaced with fresh gastric cancer organoid complete medium for continued culture.

### 2.5 Western blot

Proteins were extracted via RIPA lysis buffer (CWBIO, Beijing, China) supplemented with protease inhibitors. The extracted proteins were mixed with loading buffer and denatured by heating at 100 °C for 5 min. Proteins were separated by SDS‒PAGE and then transferred onto PVDF membranes (Millipore, MA, USA). The membranes were blocked with 5 % nonfat milk at room temperature for 2 h, followed by incubation with specific primary antibodies at 4 °C overnight. The protein bands were subsequently incubated with horseradish peroxidase (HRP)-conjugated secondary antibodies, and the signals were detected via enhanced chemiluminescence (ECL) detection reagents.

### 2.6 Cell viability assay

Cell proliferation was evaluated via the Cell Counting Kit-8 (CCK-8) Cell Proliferation and Cytotoxicity Assay Kit (purchased from Biosharp) following the manufacturer's instructions. First, the cells were seeded into 96-well plates at a density of 4000 cells per well, and subsequent relevant procedures (e.g., drug treatment, incubation for a specific period to allow cell growth, as per the experimental design) were performed. After completing the predesigned treatments, the CCK-8 reagent was diluted at a ratio of 1:12 and added to each well. The plates were then incubated in a cell culture incubator for 40 min. Finally, the absorbance at 450 nm was measured via a microplate reader (Bio-Rad, USA).

**Reactive Oxygen Species (ROS) Assay** The oxidative-sensitive fluorescent probe DCF was used following the instructions provided by the manufacturer of the DCFH-DA Assay Kit (Beyotime, China). First, the cells were exposed to 10 μM DCFH-DA and incubated for 30 min under standard cell culture conditions (37 °C, 5 % CO_2_). After the incubation period, the cells were washed three times with PBS to remove excess extracellular DCFH-DA and eliminate background fluorescence interference. The increase in fluorescence intensity associated with DCFH oxidation to DCF reported by flow cytometry.

### 2.7 Immunohistochemistry (IHC)

Gastric tissues and subcutaneous tumor tissues were dewaxed by immersion in xylene and rehydrated through a graded ethanol series. Antigen retrieval was performed using either citric acid or EDTA, following the instructions provided by the primary antibody manufacturer, with the conditions of microwaving at high power for 10 min. Endogenous peroxidase activity was blocked by incubating the sections with a peroxidase blocker for 15 min. The sections were subsequently incubated with the primary antibody overnight at 4 °C. The next day, the sections were first incubated with a reaction enhancer solution at room temperature for 20 min, followed by incubation with the secondary antibody for 10 min. After that, 3,3′-diaminobenzidine (DAB) staining was conducted. Finally, the cell nuclei were stained with hematoxylin. The stained sections were then dehydrated through a graded ethanol series, immersed in xylene for 20 min, and mounted with neutral balsam for microscopic observation.

### 2.8 Immunofluorescence (IF) staining

Gastric tissues and subcutaneous tumor tissues were dewaxed by immersion in xylene and rehydrated through a graded ethanol series. Antigen retrieval was carried out via the use of either citrate buffer or EDTA, in accordance with the instructions provided by the primary antibody manufacturer, under the condition of microwaving at high power for 10 min. Endogenous peroxidase activity in the tissues was inactivated with 3 % hydrogen peroxide. The cells were permeabilized with 0.5 % Triton X-100 for 10 min, followed by blocking of nonspecific antigen epitopes via incubation of the sections with 3 % bovine serum albumin (BSA) for 1 h. After the blocking step, the sections were incubated with the primary antibody overnight at 4 °C. The next day, the sections were incubated with a fluorescent secondary antibody at room temperature for 1 h. The cell nuclei were subsequently stained by incubating the sections with 4′, 6-diamidino-2-phenylindole (DAPI) for 10 min. Finally, the sections were mounted with an anti-fluorescence quenching agent to preserve the fluorescent signal.

### 2.9 Molecular docking and dynamics simulation

Virtual docking studies were performed via Molecular Operating Environment (MOE) software to analyze the intermolecular interactions between DMNQ and the predicted target. The crystal structure of the target protein was retrieved from the Protein Data Bank (PDB; http://www.rcsb.org). The resulting docking poses were visualized via PyMOL. The DMNQ-STAT3 complex obtained from docking was used as the initial structure for all-atom molecular dynamics (MD) simulations, which were conducted via YASARA software. The binding free energy between the protein and ligand was calculated via the MM/GBSA method, and alanine scanning was performed. A 60 ns MD trajectory was employed for these calculations, as longer MD simulations may compromise the accuracy of the MM/GBSA results.

### 2.10 Cellular Thermal Shift Assay (CETSA)

After drug treatment, the cells were lysed with cell lysis buffer and equally divided into nine EP tubes. Each tube was then heated at a specific temperature for 5 min. Loading buffer was subsequently added to each tube, and the samples were denatured by heating at 100 °C for 5 min to prepare for subsequent Western blot analysis.

### 2.11 Drug Affinity Responsive Target Stability (DARTS)

Gastric cancer cells (MKN-45, MFC) were washed twice with PBS and subsequently lysed with RIPA lysis buffer. The cell lysates were then aliquoted into separate tubes and incubated with various concentrations of DMNQ (MKN-45: 0, 20, 40, 60, 80 μM; MFC: 0, 6, 12, 18, 24 μM) at 37 °C for 1 h. Following DMNQ treatment, the samples were hydrolyzed with pronase E (MKN-45: 20 μg/mL; MFC: 10 μg/mL, MCE; Shanghai, China) at 37 °C for 20 min. The reaction was terminated by the addition of a complete enzyme inhibitor cocktail. Finally, the samples were processed for subsequent Western blot analysis.

### 2.12 CUT&Tag

CUT&Tag was performed via the Hyperactive Universal CUT&Tag Assay Kit for Illumina (Vazyme Biotech, Cat. No. TD903) following the manufacturer's protocol. Lysates of MKN-45 cells with FLAG-tagged STAT3 overexpression were incubated with 10 μL of prewashed ConA beads and 5 μg of FLAG-tag antibody. The DNA was subsequently fragmented and extracted for amplification. After amplification, all the libraries were sequenced via the Illumina HiSeq X Ten platform, and the bigWig tracks were visualized via the Integrative Genomics Viewer (IGV) browser.

### 2.13 Chromatin immunoprecipitation (ChIP) assay

Chromatin immunoprecipitation (ChIP) was conducted via the SimpleChIP® Enzymatic Chromatin IP Kit (CST, MA, USA) according to the manufacturer's recommended protocol. The DNA and proteins were cross-linked to form stable complexes. The chromatin was subsequently fragmented into small segments via sonication to ensure efficient antibody binding. Anti-STAT3 antibody and anti-Flag antibody (ABclonal, Wuhan, China) were then employed to specifically capture the target DNA fragments, which were further purified to remove impurities prior to PCR amplification. The PCR-amplified products were separated and visualized via agarose gel electrophoresis. The sequences of the primers used for ChIP-qPCR are listed in Table S2.

### 2.14 Isolation and Activation of Peripheral Blood Mononuclear Cells (PBMCs) and coculture Assay

PBMCs were isolated from the peripheral blood of healthy human donors. The PBMCs were cultured for 72 h in medium supplemented with 10 % FBS, 300 U/mL IL-2, 10 μg/mL human CD3 monoclonal antibody (mAb), and 10 μg/mL human CD28 mAb to induce activation. Activated PBMCs were then mixed with MKN-45 cells at a ratio of 5:1 in 6-well plates and cocultured for 72 h. Following coculture, the proportion of CD8^+^ T cells (CD3^+^CD8^+^) among PBMCs was analyzed via a FACScan flow cytometer (Becton Dickinson). Additionally, the apoptotic rate of MKN-45 cells was detected via an apoptosis detection kit.

### 2.15 Animal experiments

Specific pathogen-free (SPF) 615 mice and C57BL/6J-IL6st^em1(Y757F^–^V760A) Smoc^ mice were housed in SPF-grade animal facilities. The standardized housing environment included an ambient temperature of 25 ± 2 °C, 40–60 % relative humidity, and a 12-h light/dark cycle. The mice were provided with adequate water and food.

We established a subcutaneous GC animal xenograft model to evaluate carcinogenicity in vivo. GC cells (2 × 10^6^) were injected subcutaneously into the axilla of 5-week-old 615 mice (Jinan Xingkang Biotechnology), with 6 mice in each group. For the animal experiments, the siRNAs were modified with 5′-cholesterol conjugation plus 2′-*O*-methylation (2′-OMe) and phosphorothioate (PS) linkages, and then administered via direct intratumoral injection at a dose of 2 nmol per injection site. The injections were repeated once every 3–4 days for a total of 2 administrations. Tumor growth was observed every 3 days, and the mice were culled after 18 days. The tumor volume was calculated as volume = (width^2^ × length × 0.5).

For one group of C57BL/6J-IL6st^em1(Y757F^–^V760A)Smoc^ mice, treatment with DMNQ was initiated at Week 13 of the experiment. DMNQ was administered via intraperitoneal injection at a dose of 20 mg/kg three times per week for a continuous 4-week period. At week 20, the mice were euthanized via carbon dioxide inhalation. Subsequent procedures included assessing the growth of gastric cancer tissues; collecting and fixing visceral tissues (including heart, thymus, lung, liver, spleen, and kidney); and analyzing the biological toxicity of DMNQ through hematoxylin‒eosin (HE) staining. For another group of C57BL/6J-IL6st^em1(Y757F^–^V760A)Smoc^ mice, treatments were started at Week 14, with three intervention arms: single-agent DMNQ, single-agent αPD-L1, and the combination of DMNQ and αPD-L1. The administration protocol for the DMNQ was consistent with that described above. αPD-L1 was delivered via tail vein injection at a dose of 20 mg/kg, twice per week, for a total of 5 weeks. All the mice in this group were also euthanized via carbon dioxide inhalation at week 20, after which gastric cancer tissue growth was assessed to evaluate the therapeutic efficacy of the different regimens.

### 2.16 Statistical analysis

All the quantitative data are presented as the means ± standard deviations (SDs). Statistical analyses were performed via GraphPad Prism 8.0 software. For comparisons between two groups, an unpaired Student's *t*-test was applied; for comparisons among three or more groups, one-way analysis of variance (one-way ANOVA) followed by an appropriate post hoc test was used. A value of P < 0.05 was considered statistically significant. All experiments were independently repeated at least 3 times to ensure reproducibility. (∗*P* < 0.05, ∗∗*P* < 0.01, ∗∗∗*P* < 0.001, and ∗∗∗∗*P* < 0.0001).

## 3 Results

### 3.1 The ROS activator DMNQ induces ferroptosis in gastric cancer cells

**Fig. 1:**
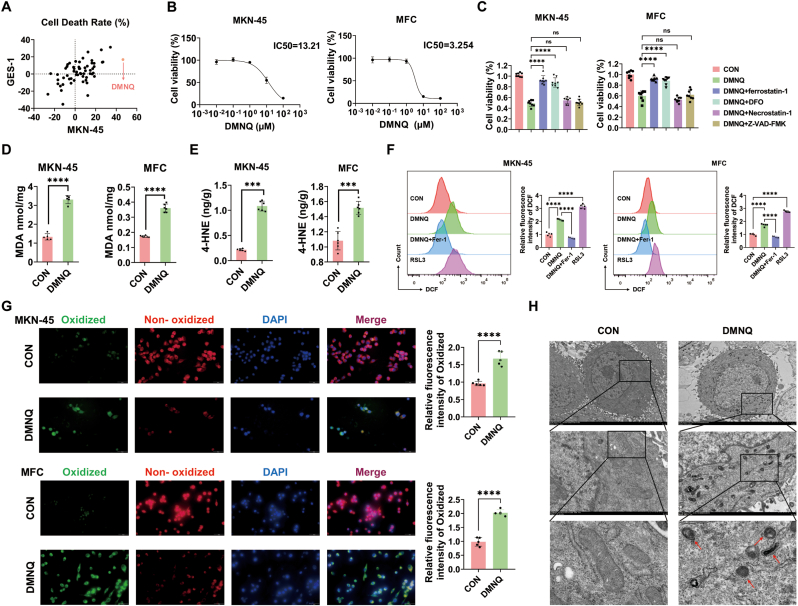
**DMNQ promotes ferroptosis in gastric cancer cells** **A** Cell death rates of gastric cancer cells and normal gastric epithelial cells after treatment with small-molecule compounds. **B** DMNQ IC50 values in MKN-45 and MFC cells were measured by the CCK-8 assay. **C** OD values of MKN-45 and MFC cells treated with DMNQ alone or in combination with Fer-1, DFO, Nec-1, or Z-VAD for 48 h. **D** MDA content in cells detected 48 h after DMNQ treatment. **E** 4-HNE content in cells detected 48 h after DMNQ treatment**. F** DCF levels detected by flow cytometry 48 h after DMNQ treatment. **G** Intracellular lipid peroxide levels observed under a fluorescence microscope: cells were treated with DMNQ for 48 h and then incubated with the C11 BODIPY 581/591 probe. **H** Mitochondrial morphology in MKN-45 cells was observed by electron microscopy 48 h after DMNQ treatment. ∗*p* < 0.05, ∗∗*p* < 0.01, ∗∗∗*p* < 0.001, ∗∗∗∗*p* < 0.0001.

To identify compounds with proferroptosis activity through the induction of ROS in gastric cancer cells, we investigated the antitumor effects of 59 natural small-molecule compounds from a commercial library associated with ROS on gastric cancer cell lines (MKN-45) and normal gastric epithelial cell lines (GES-1). Our results demonstrated that at equivalent concentrations, DMNQ significantly inhibited the proliferation of gastric cancer cells (MKN-45) but exhibited minimal toxicity in normal gastric epithelial cells (GES-1) (Fig. 1A). We selected human gastric cancer cells (MKN-45) and mouse gastric cancer cells (MFCs) for subsequent experiments. Using the Cell Counting Kit-8 (CCK-8) assay, we determined the half-maximal inhibitory concentration (IC50) of DMNQ in both cell lines (Fig. 1B). To establish the association between DMNQ-induced cell death and ferroptosis, we observed that ferroptosis inhibitors (ferrostatin-1 and DFO) effectively attenuated DMNQ-induced cytotoxicity, whereas apoptosis (Z-VAD-FMK) and necrosis (Necrostatin-1) inhibitors had no protective effects (Fig. 1C). Further investigation of ferroptosis-related markers revealed that DMNQ treatment significantly elevated malondialdehyde (MDA) and 4-hydroxynonenal (4-HNE) levels in gastric cancer cells (Fig. 1D–E). Flow cytometry analysis demonstrated a marked increase in DCF production following DMNQ treatment, with the Fer-1 group serving as the negative control and the RSL3 group as the positive control (Fig. 1F). Immunofluorescence analysis via BODIPY 581/591C11 confirmed increased lipid peroxidation in DMNQ-treated cells (Fig. 1G). Transmission electron microscopy (TEM) revealed characteristic morphological changes associated with ferroptosis, including mitochondrial shrinkage and crista reduction (Fig. 1H). Collectively, these findings provide compelling evidence that DMNQ induced ferroptosis in gastric cancer cells through ROS-mediated lipid peroxidation.

### 3.2 DMNQ binds to the SH2 domain of STAT3 and inhibits its phosphorylation

**Fig. 2:**
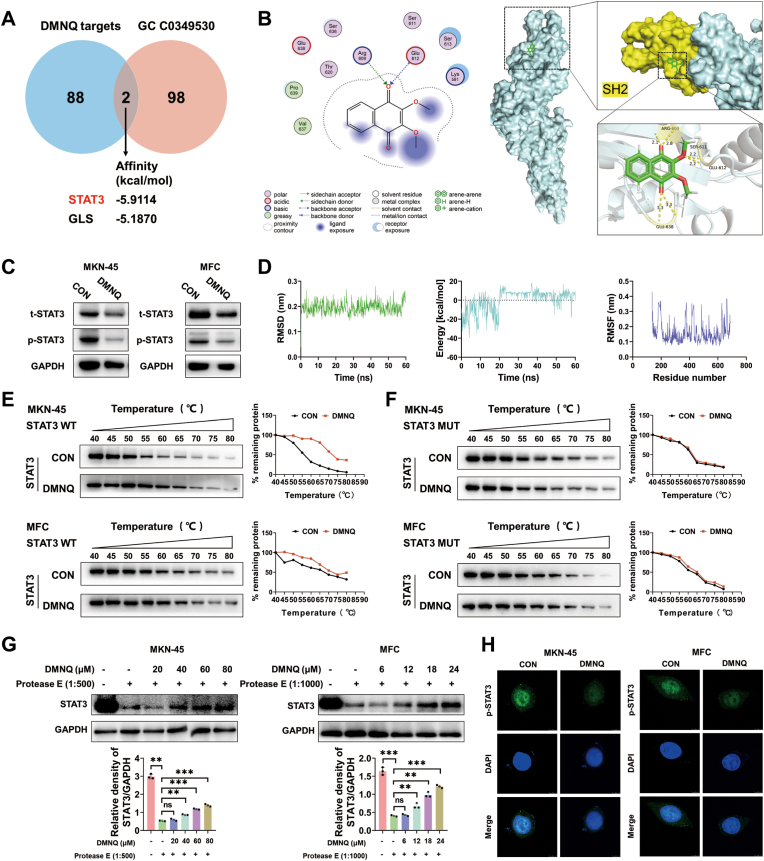
**DMNQ binds to the SH2 domain of STAT3 and inhibits its phosphorylation** **A** Venn diagram of DMNQ targets screened from the SuperPred database and gastric cancer therapeutic targets (GC 0349530). **B** Molecular docking results between DMNQ and its potential target STAT3 were obtained via MOE and PyMOL. **C** Western blot (WB) analysis of tSTAT3 and pSTAT3 levels in gastric cancer cells treated with DMNQ. **D** Molecular dynamics simulation of the DMNQ-STAT3 complex were performed via Yasara software. **E** After 48 h of DMNQ treatment, a CETSA assay was conducted; the protein stability of STAT3 was examined via WB at 40–80 °C. **F** The STAT3 protein was mutated (S611A/E612A/S613A), followed by 48 h of DMNQ treatment. A CETSA assay was then performed, and the protein stability of STAT3 was examined via WB at 40–80 °C. **G** Cell lysates were incubated with DMNQ at different concentrations and digested with pronase E, and the reaction was terminated by adding the corresponding enzyme inhibitor. The protein level of STAT3 was detected by WB. **H** After 48 h of DMNQ treatment, the level of pSTAT3 in gastric cancer cells was detected by immunofluorescence. ∗*p* < 0.05, ∗∗*p* < 0.01, ∗∗∗*p* < 0.001, ∗∗∗∗*p* < 0.0001.

To identify the target of DMNQ in gastric cancer cells, we first screened potential targets of DMNQ via the SuperPred database and intersected them with known therapeutic targets for gastric cancer. This analysis identified STAT3 and GLS as candidate targets. STAT3 exhibited a stronger molecular binding affinity to DMNQ than GLS did (Fig. 2A). Molecular docking results demonstrated that DMNQ directly bound to residues 611–613 within the SH2 domain of STAT3 (Fig. 2B). Western blot (WB) assays confirmed that DMNQ reduced total STAT3 levels in gastric cancer cells and, more prominently, inhibited STAT3 phosphorylation (Fig. 2C). Subsequent molecular dynamics (MD) simulations were performed to evaluate the stability of the DMNQ-STAT3 interaction. The root mean square deviation (RMSD) of DMNQ-STAT3 fluctuated within 0.15 nm, indicating a stable and compact DMNQ-STAT3 complex. Equilibrium trajectories were selected to simulate protein‒ligand interactions, and the root mean square fluctuation (RMSF) of most amino acid residues in the binding pocket was within 0.36 nm, confirming the structural stability of the binding pocket during dynamic simulation. The energy curve fluctuated significantly within the first 20 ns but gradually stabilized thereafter, with fluctuations controlled within 10 kcal/mol. This indicated that the system achieved thermodynamic equilibrium after 20 ns, reflecting dynamic stability in both the energy and conformation of the simulation system (Fig. 2D). A CETSA was used to assess the thermal stability of STAT3 following DMNQ binding. DMNQ significantly increased the thermal stability of STAT3 in gastric cancer cells (Fig. 2E), whereas this effect was abrogated when STAT3 was mutated at residues S611A/E612A/S613A (Fig. 2F). Additionally, the binding between DMNQ and STAT3 was validated via a drug affinity responsive target stability (DARTS) assay (Fig. 2G). Immunofluorescence staining revealed that DMNQ inhibited the nuclear localization of phosphorylated STAT3 (Fig. 2H). Collectively, these findings provided robust evidence that DMNQ targeted the SH2 domain of STAT3 and suppressed its phosphorylation activity.

### 3.3 DMNQ attenuates gastric cancer progression by inhibiting STAT3 phosphorylation in vivo

**Fig. 3:**
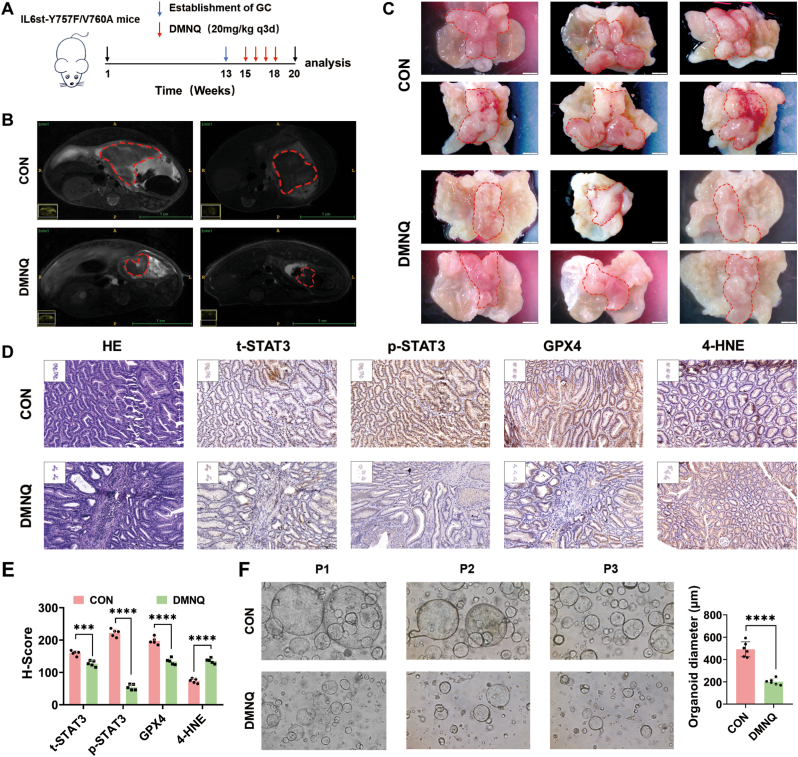
**In vivo, DMNQ inhibits STAT3 phosphorylation and attenuates GC progression** **A** Schematic diagram of gastric cancer formation in C57BL/6J-IL6st^em1(Y757F^–^V760A)Smoc^ mice and the DMNQ treatment protocol. **B** Gastric cancer size in mice were observed by magnetic resonance imaging (MRI). **C** Mice were anesthetized and sacrificed at 20 weeks of age; the size of the gastric tumors was observed and photographed under a stereomicroscope. **D‒E** Expression levels of tSTAT3, pSTAT3, GPX4, and 4-HNE were determined via immunohistochemistry (IHC), and their scores were quantified. **F** Human gastric cancer organoids were treated with DMNQ for 48 h; the organoids were photographed, and their sizes were quantified. ∗*p* < 0.05, ∗∗*p* < 0.01, ∗∗∗*p* < 0.001, ∗∗∗∗*p* < 0.0001.

To establish a gastric cancer model, we generated C57BL/6 mice with an IL6st gene mutation that constitutively activated STAT3 signaling (C57BL/6J-IL6st^em1(Y757F^– ^V760A)Smoc^, IL6st-Y757F–V760A). The transgenic mice began to develop gastric cancer spontaneously beginning at week 13 (Supplementary Fig. 1A–B). To verify the therapeutic effect of DMNQ on gastric cancer in vivo, DMNQ was administered via intraperitoneal injection at a dose of 20 mg/kg for 4 weeks (Fig. 3A). Gastric magnetic resonance imaging and stereomicroscopy revealed that DMNQ significantly inhibited the progression of gastric cancer (Fig. 3B–C). Moreover, pathological examination of major organs revealed no signs of toxicity associated with DMNQ treatment (Supplementary Fig. 1C). The immunohistochemistry (IHC) results demonstrated that after DMNQ treatment, the expression of total STAT3 in gastric cancer tissues was reduced, the expression of phosphorylated STAT3 (p-STAT3) and the ferroptosis-inhibiting molecule GPX4 was significantly decreased, and the expression of 4-HNE was increased (Fig. 3D–E). Furthermore, by establishing organoid models from clinical gastric cancer patients, we confirmed that DMNQ significantly inhibited the growth rate of gastric cancer organoids (Fig. 3F). These results indicated that DMNQ inhibited gastric cancer growth by suppressing STAT3 phosphorylation.

### 3.4 SLC1A4, a transcriptional target of STAT3, inhibits ferroptosis in gastric cancer cells by modulating cysteine transport

**Fig. 4:**
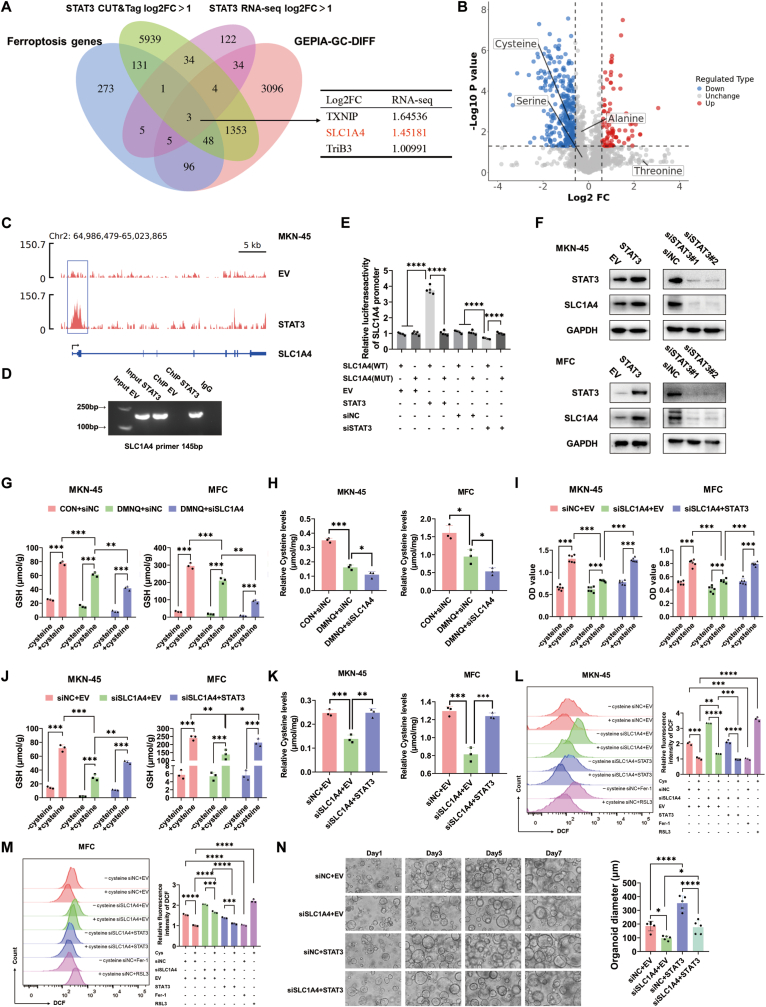
**SLC1A4, a transcriptional target of STAT3, inhibits ferroptosis in gastric cancer cells by modulating cysteine transport** **A** Venn diagram of differentially expressed genes from CUT&Tag, RNA-Seq (MKN-45 STAT3 vs EV), ferroptosis-related genes, and genes associated with survival differences in gastric cancer patients. **B** Volcano plot of differentially abundant metabolites obtained from untargeted metabolomic sequencing of gastric cancer tissues in DMNQ-treated mice. **C** IGV visualization of SLC1A4 enrichment in the promoter regions of MKN-45 E V and STAT3. **D** ChIP assay to detect whether SLC1A4 binds to the STAT3 promoter region. **E**. Transcriptional activity of SLC1A4 was measured by the luciferase reporter system. Reporter activity is reported as the fold activation relative to Renilla luciferase activity. **F** WB analysis of the expression trend of SLC1A4 following changes in STAT3 protein expression. **G** Intracellular GSH content was detected in cells treated with DMNQ and siSLC1A4 under normal medium and cysteine-depleted medium conditions. **H** Intracellular cysteine content was detected in cells treated with DMNQ and siSLC1A4. **I** Cell viability changes were detected by the CCK-8 assay in cells treated with siSLC1A4 and STAT3 under normal medium and cysteine-depleted medium conditions. **J** Intracellular GSH content was detected in cells treated with siSLC1A4 and STAT3 in normal medium and cysteine-depleted medium. **K** Intracellular cysteine content was detected in cells treated with siSLC1A4 and STAT3. **L‒M** Intracellular DCF levels were detected via flow cytometry in cells treated with siSLC1A4 and STAT3 under normal medium and cysteine-depleted medium conditions. **N** Human gastric cancer organoids were treated with siSLC1A4 and STAT3; the organoids were photographed, and their sizes were quantified. ∗*p* < 0.05, ∗∗*p* < 0.01, ∗∗∗*p* < 0.001, ∗∗∗∗*p* < 0.0001.

As a transcription factor, STAT3 regulates downstream target genes to mediate cellular processes. To identify the downstream targets of STAT3, we established stable STAT3-overexpressing MKN-45 and MFC cell lines (Supplementary Fig. 2). Target genes were screened via CUT&Tag and RNA-Seq analyses, and by intersecting these genes with ferroptosis-related genes and differentially expressed genes from gastric cancer patients, three potential downstream target genes of STAT3 (TXNIP, SLC1A4, and TriB3) were identified (Fig. 4A). Aberrant metabolic cues critically impact ROS relevant to ferroptosis in cancer cells. To clarify the specific mechanism by which DMNQ targets STAT3 to modulate ferroptosis, we further performed untargeted metabolomic sequencing on gastric cancer tissues from DMNQ-treated mice. The results revealed that cysteine expression decreased after DMNQ treatment (Fig. 4B). Notably, SLC1A4 is a well-characterized cysteine transporter. Thus, we hypothesized that STAT3 transcriptionally regulates SLC1A4 expression to control cysteine uptake in gastric cancer cells, thereby inhibiting ferroptosis.

The IGV results of CUT&Tag revealed significant enrichment of SLC1A4 at the STAT3 promoter region (Fig. 4C). Chromatin immunoprecipitation (ChIP) assays demonstrated that STAT3 directly bound to the promoter of SLC1A4 to regulate its expression (Fig. 4D). The dual-luciferase assay was conducted according to the binding site and the co-transfection of STAT3 overexpression and SLC1A4-promoter WT markedly induced the luciferase activity, while STAT3 silencing had the opposite effect, indicating the targeted binding between STAT3 and SLC1A4 (Fig. 4E).Consistent with the sequencing results, WB analysis revealed that STAT3 overexpression significantly increased SLC1A4 expression. On the other hand, knocking down STAT3 in gastric cancer cells led to opposite results (Fig. 4F).

To investigate the role of DMNQ-mediated STAT3‒SLC1A4 cysteine transport in gastric cancer cell ferroptosis, we conducted functional experiments. Supplementation with cysteine in cysteine-depleted medium restored intracellular glutathione (GSH) levels, but this restoration was attenuated in DMNQ-treated gastric cancer cells and further impaired when cysteine was combined with siSLC1A4 (Fig. 4G). Direct measurement of intracellular cysteine levels confirmed that DMNQ inhibits cysteine intracellular transport by targeting SLC1A4 (Fig. 4H). Cysteine rescue experiments further revealed that SLC1A4, which is regulated by STAT3, inhibits gastric cancer cell growth through cysteine transport (Fig. 4I). Moreover, siSLC1A4 suppressed intracellular GSH synthesis and cysteine transport, which was reversed by STAT3 overexpression (Fig. 4J–K). Flow cytometry analysis revealed that siSLC1A4 increased DCF levels by reducing cysteine uptake in gastric cancer cells, while STAT3 overexpression reversed this effect, with the Fer-1 group serving as the negative control and the RSL3 group as the positive control (Fig. 4L–M). Organoid models confirmed that siSLC1A4 inhibited the growth of gastric cancer organoids, and this inhibition was abrogated by STAT3 overexpression (Fig. 4N). Collectively, these experiments demonstrated that DMNQ promoted ferroptosis in gastric cancer cells by targeting the STAT3/SLC1A4 axis to inhibit cysteine transport.

### 3.5 In vivo, SLC1A4 transports cysteine under the regulation of STAT3 to promote gastric cancer cell growth

**Fig. 5:**
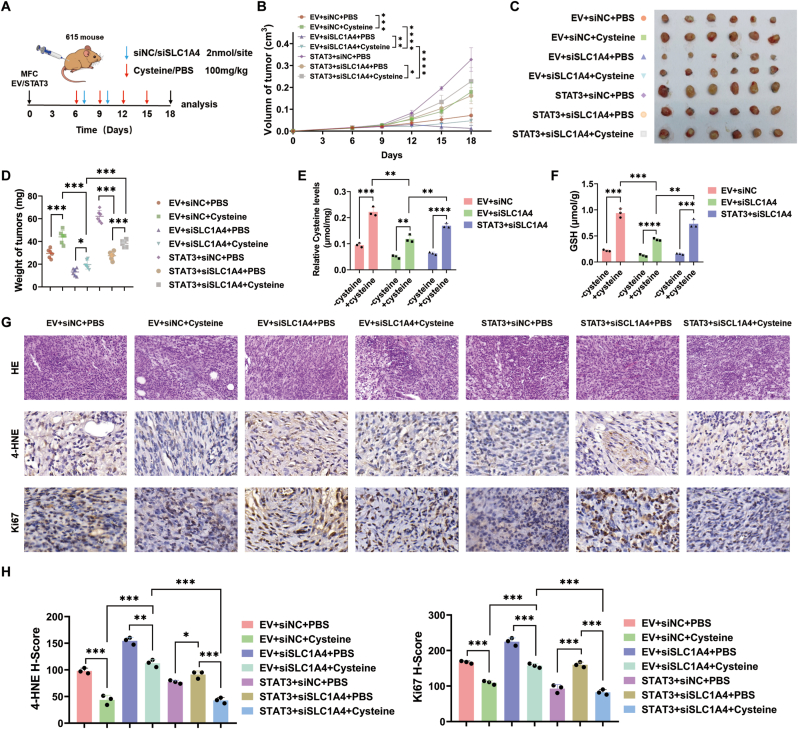
**In vivo, SLC1A4 transports cysteine under the regulation of STAT3 to promote gastric cancer cell growth** **A** Flowchart of the animal experimental procedures. **B** For the 615 mice bearing subcutaneous tumors, the tumor size was measured starting from the 6th day, with measurements taken every 3 days until the 18th day. **C‒D** On the 18th day, the mice were anesthetized and sacrificed; the tumors were collected, photographed, and weighed. **E‒F** Cysteine and GSH contents in tumors were measured. **G-H** The levels of 4-HNE and Ki67 in xenograft tumors were determined by IHC, and the staining scores were quantified. ∗*p* < 0.05, ∗∗*p* < 0.01, ∗∗∗*p* < 0.001, ∗∗∗∗*p* < 0.0001.

To evaluate the role of targeting SLC1A4-mediated cysteine transport in delaying gastric cancer growth in vivo, we subcutaneously injected MFC cells into 5-week-old 615 strain mice. Subsequently, intraperitoneal injection of cysteine significantly promoted tumor growth, whereas intratumoral injection of siSLC1A4 delayed tumor growth and attenuated the tumor-promoting effect of cysteine. The overexpression of STAT3 reversed the tumor growth inhibition induced by SLC1A4 suppression and partially restored cysteine-mediated tumor promotion (Fig. 5A–D). Analysis of tumor tissues revealed that cysteine supplementation enhanced intracellular GSH synthesis, whereas siSLC1A4 inhibited both intracellular GSH synthesis and cysteine transport. These inhibitory effects were reversed by combined STAT3 overexpression (Fig. 5E–F). Additionally, immunohistochemical (IHC) staining of subcutaneous tumors revealed that cysteine supplementation reduced the expression of the ferroptosis marker 4-HNE and the proliferation marker Ki67 in tumor tissues. In contrast, intratumoral siSLC1A4 injection increased the expression of 4-HNE and Ki67, and this effect was attenuated by STAT3 overexpression (Fig. 5G–H).

### 3.6 SLC1A4 promotes tumor immunosuppression by inhibiting cysteine uptake in gastric cancer cells

**Fig. 6:**
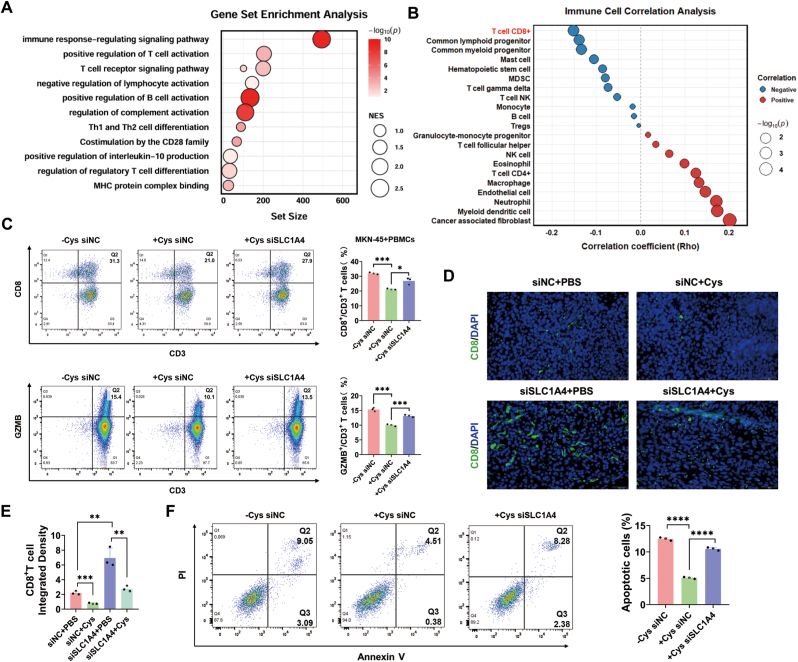
**SLC1A4 promotes tumor immunosuppression by inhibiting cysteine uptake in gastric cancer cells** **A** Enrichment analysis of SLC1A4-related pathways in gastric cancer tissues via the TCGA database. **B** Correlations between SLC1A4 expression and immune cells in the gastric cancer microenvironment were analyzed via the TIMER 2.0 database (http://timer.comp-genomics.org/). **C** PBMCs were cocultured with tumor cells; the effects of cysteine and siSLC1A4 on the proportions of CD8^+^T cells and GZMB^+^T cells were analyzed by flow cytometry. **D-E** Immunofluorescence showing the effects of cysteine and siSLC1A4 on CD8^+^T-cell infiltration in xenograft tumors. **F** PBMCs were cocultured with tumor cells; the effects of cysteine and siSLC1A4 on the tumor-killing ability of CD8^+^T cells were analyzed via flow cytometry. ∗*p* < 0.05, ∗∗*p* < 0.01, ∗∗∗*p* < 0.001, ∗∗∗∗*p* < 0.0001.

Recent studies have demonstrated regulatory relationships between amino acids and the TME. We observed that elevated SLC1A4 expression was negatively correlated with immune pathways (Fig. 6A) and with CD8^+^ T lymphocyte infiltration (Fig. 6B). We thus hypothesized that SLC1A4 regulated CD8^+^ T cells in the TME via cysteine residues. MKN-45 cells were co-cultured with peripheral blood mononuclear cells (PBMCs) from healthy volunteers. As expected, cysteine deprivation resulted in a decreased proportion of CD8^+^ T cells and reduced GZMB levels. Furthermore, SLC1A4 knockdown attenuated the cysteine-induced increases in the proportion of CD8^+^ T cells and GZMB levels (Fig. 6C). Immunofluorescence analysis of mouse subcutaneous tumors revealed that cysteine supplementation reduced the infiltration ratio of CD8^+^ T cells in tumor tissues, whereas SLC1A4 knockdown increased CD8^+^ T cell infiltration by regulating cysteine (Fig. 6D–E). Coculture experiments with CD8^+^ T cells and MKN-45 cells revealed that cysteine deprivation increased the cytotoxic capacity of CD8^+^ T cells against tumor cells, whereas SLC1A4 knockdown reversed the cysteine-induced impairment of CD8^+^ T cell cytotoxicity (Fig. 6F). Collectively, these findings demonstrated that inhibiting SLC1A4 expression could promote CD8^+^ T-cell infiltration in gastric cancer tissues and enhance their cytotoxicity against gastric cancer cells by reducing cysteine uptake.

### 3.7 In vivo, inhibiting SLC1A4 expression enhances the therapeutic efficacy of αPD-L1 by regulating cysteine levels

**Fig. 7:**
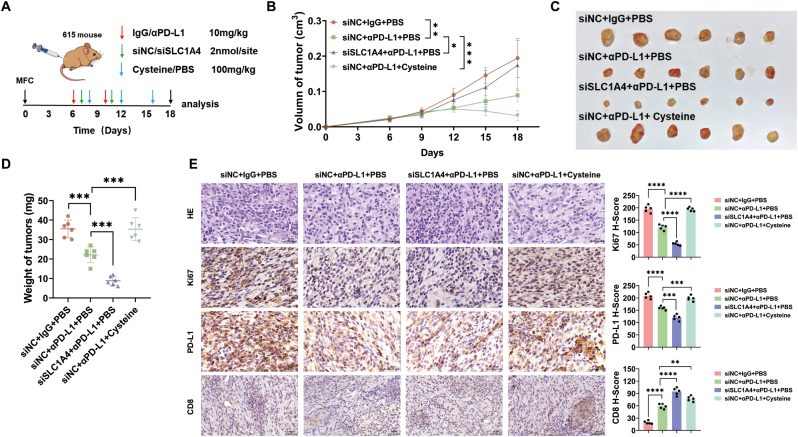
**In vivo, inhibiting SLC1A4 expression enhances the therapeutic efficacy of αPD-L1 by regulating cysteine levels** **A** Flowchart of the animal experimental procedures. **B** For 615 mice with subcutaneous tumors, tumor size was measured starting from the 6th day after tumor implantation, with measurements taken every 3 days until the 18th day. **C‒D** On the 18th day, the mice were anesthetized and sacrificed; the tumors were harvested, photographed, and weighed. **E** The levels of Ki67, PD-L1, and CD8 in xenograft tumors were determined by IHC, and the staining scores were quantified. ∗*p* < 0.05, ∗∗*p* < 0.01, ∗∗∗*p* < 0.001, ∗∗∗∗*p* < 0.0001.

To evaluate the role of targeting SLC1A4-mediated cysteine transport in enhancing αPD-L1 immunotherapy for gastric cancer, we subcutaneously injected MFC cells into 5-week-old 615 strain mice. Intravenous injection of αPD-L1 slowed the growth of gastric cancer, whereas intratumoral injection of siSLC1A4 significantly enhanced the tumor-inhibitory effect of αPD-L1. Conversely, cysteine supplementation attenuated the therapeutic efficacy of αPD-L1 (Fig. 7A–D). Consistent with these findings, the immunohistochemical results revealed that αPD-L1 treatment reduced the expression of Ki67 and PD-L1 in gastric cancer cells and increased CD8^+^ T cell infiltration in tumor tissues. Compared with αPD-L1 monotherapy, siSLC1A4 further decreased Ki67 and PD-L1 expression and further increased CD8^+^ T cell infiltration. In contrast, cysteine supplementation increased Ki67 and PD-L1 expression and reduced CD8^+^ T cell infiltration compared with those in the αPD-L1 monotherapy group (Fig. 7E). These results suggested that siSLC1A4 enhanced the in vivo therapeutic efficacy of αPD-L1 against gastric cancer by regulating cysteine levels.

### 3.8 Combined with the DMNQ significantly enhances the immunotherapeutic efficacy of αPD-L1 in gastric cancer

**Fig. 8:**
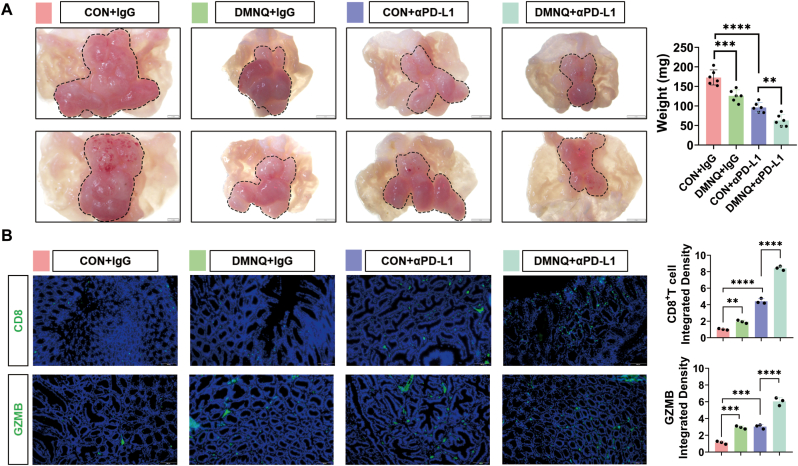
**Combined with the DMNQ significantly enhances the immunotherapeutic efficacy of αPD-L1 in gastric cancer** **A** Tumor sizes of orthotopic gastric cancer model mice after treatment with DMNQ or αPD-L1. **B** Fluorescence intensities of CD8 and GZMB in different gastric cancer tissue samples were determined by immunofluorescence, followed by quantification and statistical analysis. ∗*p* < 0.05, ∗∗*p* < 0.01, ∗∗∗*p* < 0.001, ∗∗∗∗*p* < 0.0001.

Having clarified the potential of cysteine restriction in gastric cancer therapy, we further evaluated the efficacy of DMNQ in reversing αPD-L1 resistance in a mouse orthotopic gastric cancer model. Thirteen-week-old C57BL/6J-IL6st^em1 (Y757F^–^V760A) Smoc^ mice were treated with DMNQ, αPD-L1 alone, or their combination. Gastric tissues were examined at 20 weeks of age, and the results revealed that the tumor size in the DMNQ and αPD-L1 combination group was significantly smaller than that in the αPD-L1 monotherapy group, indicating that the combined treatment of DMNQ and αPD-L1 exerted a superior therapeutic effect to that of αPD-L1 alone (Fig. 8A). Immunofluorescence analysis revealed that the infiltration ratio of CD8^+^ T cells and the expression of GZMB in the DMNQ and αPD-L1 groups were significantly greater than those in the αPD-L1 monotherapy group (Fig. 8B). These findings suggested that combining DMNQ with αPD-L1 could significantly increase the immunotherapeutic efficacy of αPD-L1 against gastric cancer.

## 4 Discussion

Ferroptosis has emerged as a novel therapeutic strategy in oncology, demonstrating unique advantages in addressing issues of low targeting efficiency and drug resistance in cancer treatment [19]. Moreover, immunotherapy has revolutionized the landscape of cancer therapy [27]. In patients with gastric cancer, immunotherapy has become a first-line option for advanced patients, significantly prolonging survival in a subset of patients and representing a breakthrough in disease management [28]. The combination of ferroptosis inducers with immune checkpoint inhibitors such as PD-1/PD-L1 blockers has synergistic effects on cancer, directly killing tumor cells while activating immune-mediated tumor attack [29]. Our study revealed that DMNQ targeted the SH2 domain of STAT3, inhibited its phosphorylation, and subsequently downregulated the transcription of SLC1A4. As a cysteine transporter, SLC1A4 suppression reduced cysteine uptake in gastric cancer cells, leading to excessive ROS accumulation and a decrease in PD-L1 expression, ultimately enhancing the efficacy of ICIs.

As a key transcriptional regulator, STAT3 not only significantly impacts the survival of tumor cells but also shapes an immunosuppressive TME, contributing to resistance to immunotherapy [30,31]. Consequently, inhibiting STAT3 activity has emerged as an effective therapeutic strategy for treating tumors. Natural products, including both direct and indirect inhibitors, have emerged as rich sources of STAT3 inhibitors. Direct natural inhibitors, such as silibinin [32], pretrichodermamide B, sinomenine [33], and galiellalactone [34], physically bind SH2 or DNA-binding domains, effectively blocking dimerization and phosphorylation of STAT3. A number of these agents are currently under preclinical and clinical investigation for various human cancers [35], highlighting the translational potential of STAT3-targeted therapies. In our study, we discovered that DMNQ targeted the SH2 domain of STAT3. Importantly, DMNQ induced ferroptosis in gastric cancer cells through ROS-mediated lipid peroxidation both in vitro and in vivo. Our study emphasized the remarkable efficacy and therapeutic potential of DMNQ as a treatment for gastric cancer.

Different amino acids act as major regulators that can either “brake” or “accelerate” ferroptosis, primarily by influencing two central cellular redox systems: the glutathione (GSH) system and the phospholipid peroxide repair system [36]. We discovered that DMNQ decreased the cysteine content in gastric cancer cells, leading to intracellular ROS accumulation and ferroptosis. Intriguingly, STAT3 transcriptionally regulates the expression of the membrane transporter SLC1A4, thereby promoting cysteine uptake in tumor cells. Cysteine plays an essential role in this process as a substrate for GSH synthesis [37]. Moreover, under cysteine-deficient conditions, serine and methionine can be converted into cysteine to supply substrates for GSH synthesis, thereby resisting ferroptosis [36,38]. A recent study revealed that pancreatic ductal adenocarcinoma (PDAC) cells can modulate the cysteine transporter SLC7A11 via autophagy [39]. However, how cysteine uptake regulation influences ferroptosis in gastric cancer remains unexplored. Through CUT&Tag and RNA-seq analyses, SLC1A4 was identified as a downstream target of STAT3. Furthermore, SLC1A4 knockdown inhibited the growth of gastric cancer, and this inhibition was abrogated by STAT3 overexpression. By employing gastric cancer cells, organoids, and mouse models, we confirmed SLC1A4 transported cysteine under the regulation of STAT3 to increased GSH levels and thereby inhibited ferroptosis in gastric cancer cells.

Excessive ROS in tumor cells can induce ferroptosis, leading to the release of tumor-associated antigens and damage-associated molecular patterns (e.g., ATP and HMGB1) [40,41]. These signals enhance the recognition, phagocytosis, and processing of tumor antigens by dendritic cells, which in turn present tumor antigens to CD8^+^ T lymphocytes, activating cytotoxic T cells and promoting immunogenic cell death. This pro-oxidant therapy combined with immune checkpoint inhibitors has been shown to convert immunologically "cold" tumors into "hot" ones [[42], [43], [44]]. In this study, we demonstrated that DMNQ inhibited cysteine uptake by targeting the STAT3/SLC1A4 axis. Reportedly, STAT3 upregulates PD-L1 expression and exacerbates T cell exhaustion [[45], [46], [47]]. After the inhibition of STAT3, the expression of PD-L1 in gastric cancer cells was downregulated. We further reported that SLC1A4-mediated cysteine uptake decreased which could promote the infiltration of CD8^+^ T cells. Recently, cysteine metabolism was found to be responsible for various immune responses in cancer cells. In highly aggressive tumors such as pancreatic cancer and triple-negative breast cancer, cysteine metabolism is markedly upregulated, contributing to an immunosuppressive "cold" tumor microenvironment. Restricting cysteine levels in triple-negative breast cancer remodels tumor metabolic pathways and induces immunogenic cell death, significantly suppressing tumor growth and eliciting durable antitumor immune memory [48,49]. Cysteine deprivation also promotes M1-like polarization of tumor-associated macrophages and activates cytotoxic T lymphocytes, thereby reversing the immunosuppressive tumor microenvironment [50]. Our study demonstrated that DMNQ inhibited cysteine uptake in tumor cells, inducing ferroptosis and concurrently reversing the immunosuppressive tumor microenvironment through dual mechanisms—promoting ferroptosis and inducing cysteine deprivation—thereby enhancing the efficacy of PD-L1 blockade.

In summary, our findings highlight the anti-cancer activity of DMNQ in gastric cancer. DMNQ treatment led to ferroptosis via the STAT3/SLC1A4 axis through repressing tumor cysteine uptake, and enhanced the therapeutic effect of anti-PD-L1 immunotherapy. This work might present a novel target for new drug development in the treatment of gastric cancer.

## Declarations

All co-authors have contributed to, read and approved the final manuscript for submission and there is no financial interest to report. We certify that the manuscript is original research that has neither been published nor submitted for publication elsewhere.

## Ethics approval

This study was approved by the Medical Ethics Committee of the Central Hospital Affiliated to Shandong First Medical University.

## Data availability statement

The data that support the findings of this study are available from the corresponding author upon reasonable request.

## Funding

The study was funded by the 10.13039/501100001809National Natural Science Foundation of China (Nos. 31970728 and 82272409) and the 10.13039/501100007129Natural Science Foundation of Shandong Province (No. ZR2020LZL005), Shandong Province Medical and Health Science and Technology Project (202503030879), and the Taishan Scholar Program of Shandong (tstp20231257).

## CRediT authorship contribution statement

**Wenshuai Zhu:** Data curation, Formal analysis, Writing – original draft. **He Qi:** Data curation. **Fubo Jing:** Data curation. **Yuxuan Shi:** Formal analysis. **Yuanxin Xing:** Formal analysis. **Xiaoli Ma:** Formal analysis. **Bin Ning:** Formal analysis. **Yunshan Wang:** Conceptualization, Methodology. **Yanfei Jia:** Conceptualization, Funding acquisition, Project administration, Writing – review & editing.

## Declaration of competing interest

The authors declare that they have no known competing financial interests or personal relationships that could have appeared to influence the work reported in this paper.

## Data Availability

Data will be made available on request.

## Abbreviations

DMNQ, 2,3-dimethoxy-1,4-naphthoquinone; nti-PD-L1, anti-Programmed Death Ligand 1; TME, Tumor microenvironment; STAT3, Signal transducer and activator of transcription 3; SLC1A4, Solute carrier family 1 member 4; GPX4, Glutathione peroxidase 4; GZMB, Granzyme B; ICIs, Immune checkpoint inhibitors; ROS, Reactive oxygen species; GSH, Glutathione; CETSA, Cellular thermal shift assay; DARTS, Drug affinity responsive target stability; CUT&Tag, Cleavage under targets and tagmentation; ChIP, Chromatin Immunoprecipitation; PBMCs, Peripheral blood mononuclear cells; RMSF, Root mean square fluctuation.

## References

[1] Sundar R., Nakayama I., Markar S.R., Shitara K., van Laarhoven H.W.M., Janjigian Y.Y., Smyth E.C. (2025). Gastric cancer. Lancet (London, England).

[2] Guan W.L., He Y., Xu R.H. (2023). Gastric cancer treatment: recent progress and future perspectives. J. Hematol. Oncol..

[3] Yasuda T., Wang Y.A. (2024). Gastric cancer immunosuppressive microenvironment heterogeneity: implications for therapy development. Trends Cancer..

[4] Chen Y., Jia K., Xie Y., Yuan J., Liu D., Jiang L., Peng H., Zhong J., Li J., Zhang X., Shen L. (2025). The current landscape of gastric cancer and gastroesophageal junction cancer diagnosis and treatment in China: a comprehensive nationwide cohort analysis. J. Hematol. Oncol..

[5] Pilonis N.D., Tischkowitz M., Fitzgerald R.C., di Pietro M. (2021). Hereditary diffuse gastric cancer: approaches to screening, surveillance, and treatment. Annu. Rev. Med..

[6] Li J., Wu Z., Lin R. (2024). Impact of Helicobacter pylori on immunotherapy in gastric cancer. J. Immunother. Cancer.

[7] Zhou Q., Meng Y., Li D., Yao L., Le J., Liu Y., Sun Y., Zeng F., Chen X., Deng G. (2024). Ferroptosis in cancer: from molecular mechanisms to therapeutic strategies. Signal Transduct. Targeted Ther..

[8] Liu Y., Lu S., Wu L.L., Yang L., Yang L., Wang J. (2023). The diversified role of mitochondria in ferroptosis in cancer. Cell Death Dis..

[9] Qiu S., Zhong X., Meng X., Li S., Qian X., Lu H., Cai J., Zhang Y., Wang M., Ye Z., Zhang H., Gao P. (2023). Mitochondria-localized cGAS suppresses ferroptosis to promote cancer progression. Cell Res..

[10] Chung C.H., Lin C.Y., Chen C.Y., Hsueh C.W., Chang Y.W., Wang C.C., Chu P.Y., Tai S.K., Yang M.H. (2023). Ferroptosis signature shapes the immune profiles to enhance the response to immune checkpoint inhibitors in head and neck cancer. Adv. Science. (Weinheim, Baden-Wurttemberg, Germany).

[11] Li J., Liu J., Zhou Z., Wu R., Chen X., Yu C., Stockwell B., Kroemer G., Kang R., Tang D. (2023). Tumor-specific GPX4 degradation enhances ferroptosis-initiated antitumor immune response in mouse models of pancreatic cancer. Sci. Transl. Med..

[12] Tao Q., Liu N., Wu J., Chen J., Chen X., Peng C. (2024). Mefloquine enhances the efficacy of anti-PD-1 immunotherapy via IFN-γ-STAT1-IRF1-LPCAT3-induced ferroptosis in tumors. J. Immunother. Cancer.

[13] Zhou X., Li Y., Zhang X., Li B., Jin S., Wu M., Zhou X., Dong Q., Du J., Zhai W., Wu Y., Qiu L., Li G., Qi Y., Zhao W., Gao Y. (2024). Hemin blocks TIGIT/PVR interaction and induces ferroptosis to elicit synergistic effects of cancer immunotherapy, science China. Life Sci..

[14] Park S.Y., Kim K.Y., Gwak D.S., Shin S.Y., Jun D.Y., Kim Y.H. (2024). L-Cysteine mitigates ROS-Induced apoptosis and neurocognitive deficits by protecting against endoplasmic reticulum stress and mitochondrial dysfunction in mouse neuronal cells. Biomedicine. Pharmacotherapy = Biomedecine & Pharmacotherapie.

[15] Lin J., Rao D., Zhang M., Gao Q. (2024). Metabolic reprogramming in the tumor microenvironment of liver cancer. J. Hematol. Oncol..

[16] Zhang H., Hu Q., Zhang Y., Yang L., Tian S., Zhang X., Shen H., Shu H., Xie L., Wu D., Zhou L., Wei X., Cheng C., Jiang J., Wang H., Shen C., Kong D., Xu L. (2025). Lachnospiraceae bacterium alleviates alcohol-associated liver disease by enhancing N-acetyl-glutamic acid levels and inhibiting ferroptosis through the KEAP1-NRF2 pathway. Gut Microbes.

[17] Luo L., Wu X., Fan J., Dong L., Wang M., Zeng Y., Li S., Yang W., Jiang J., Wang K. (2024). FBXO7 ubiquitinates PRMT1 to suppress serine synthesis and tumor growth in hepatocellular carcinoma. Nat. Commun..

[18] Swanda R.V., Ji Q., Wu X., Yan J., Dong L., Mao Y., Uematsu S., Dong Y., Qian S.B. (2023). Lysosomal cystine governs ferroptosis sensitivity in cancer via cysteine stress response. Molecular cell..

[19] Wahida A., Conrad M. (2025). Decoding ferroptosis for cancer therapy. Nat. Rev. Cancer..

[20] Badgley M.A., Kremer D.M., Maurer H.C., DelGiorno K.E., Lee H.J., Purohit V., Sagalovskiy I.R., Ma A., Kapilian J., Firl C.E.M., Decker A.R., Sastra S.A., Palermo C.F., Andrade L.R., Sajjakulnukit P., Zhang L., Tolstyka Z.P., Hirschhorn T., Lamb C., Liu T., Gu W., Seeley E.S., Stone E., Georgiou G., Manor U., Iuga A., Wahl G.M., Stockwell B.R., Lyssiotis C.A., Olive K.P. (2020). Cysteine depletion induces pancreatic tumor ferroptosis in mice. Sci. (New York, N.Y.).

[21] Cramer S.L., Saha A., Liu J., Tadi S., Tiziani S., Yan W., Triplett K., Lamb C., Alters S.E., Rowlinson S., Zhang Y.J., Keating M.J., Huang P., DiGiovanni J., Georgiou G., Stone E. (2017). Systemic depletion of L-cyst(e)ine with cyst(e)inase increases reactive oxygen species and suppresses tumor growth. Nat. Med..

[22] Hofmann K., Düker M., Fink T., Lichter P., Stoffel W. (1994). Human neutral amino acid transporter ASCT1: structure of the gene (SLC1A4) and localization to chromosome 2p13-p15. Genomics..

[23] Zhang S., Huang F., Wang Y., Long Y., Li Y., Kang Y., Gao W., Zhang X., Wen Y., Wang Y., Pan L., Xia Y., Yang Z., Yang Y., Mo H., Li B., Hu J., Song Y., Zhang S., Dong S., Du X., Li Y., Liu Y., Liao W., Gao Y., Zhang Y., Chen H., Liang Y., Chen J., Weng H., Huang H. (2024). NAT10-mediated mRNA N(4)-acetylcytidine reprograms serine metabolism to drive leukaemogenesis and stemness in acute myeloid leukaemia. Nat. Cell Biol..

[24] Yang M., Huang C., Luo W., Gong P., Huang B., Yao Y. (2025). l-cysteine rescues radiation induced ferroptosis and boosts wound healing through fibroblasts proliferation and migration. Biochem. Biophys. Res. Commun..

[25] Zhou J., Tison K., Zhou H., Bai L., Acharyya R.K., McEachern D., Metwally H., Wang Y., Pitter M., Choi J.E., Vatan L., Liao P., Yu J., Lin H., Jiang L., Wei S., Gao X., Grove S., Parolia A., Cieslik M., Kryczek I., Green M.D., Lin J.X., Chinnaiyan A.M., Leonard W.J., Wang S., Zou W. (2025). STAT5 and STAT3 balance shapes dendritic cell function and tumour immunity. Nature..

[26] Li Y.J., Zhang C., Martincuks A., Herrmann A., Yu H. (2023). STAT proteins in cancer: orchestration of metabolism. Nat. Rev. Cancer.

[27] Hayday A., Dechanet-Merville J., Rossjohn J., Silva-Santos B. (2024). Cancer immunotherapy by γδ T cells. Science (New York, N.Y.).

[28] Migliore C., Fenocchio E., Giordano S., Corso S. (2025). Precision oncology in gastric cancer: shaping the future of personalized treatment. Cancer Treat Rev..

[29] Lin Y.Y., Ding J.L., Shen H.T., Lin Y.M., Adhidjaja E.C., Chang S.C. (2025). PD-1/PD-L1 cancer immunotherapeutics reshape tumor microenvironment - clinical evidence and molecular mechanisms for AI-based precision medicine. Clin. Rev. Allergy Immunol..

[30] Zou S., Tong Q., Liu B., Huang W., Tian Y., Fu X. (2020).

[31] Samad M.A., Ahmad I., Hasan A., Alhashmi M.H., Ayub A., Al-Abbasi F.A., Kumer A., Tabrez S. (2025). STAT3 signaling pathway in health and disease. MedComm..

[32] Li R., Zhou Y., Zhang X., Yang L., Liu J., Wightman S.M., Lv L., Liu Z., Wang C.Y., Zhao C. (2023). Identification of marine natural product pretrichodermamide B as a STAT3 inhibitor for efficient anticancer therapy. Marine Life Sci. Technol..

[33] Wang Y., Wu L., Cai H., Lei H., Ma C.M., Yang L., Xu H., Zhu Q., Yao Z., Wu Y. (2018). Sinomenine derivative YL064: a novel STAT3 inhibitor with promising anti-myeloma activity. Cell Death Dis..

[34] Hellsten R., Lilljebjörn L., Johansson M., Leandersson K., Bjartell A. (2019). The STAT3 inhibitor galiellalactone inhibits the generation of MDSC-Like monocytes by prostate cancer cells and decreases immunosuppressive and tumorigenic factors. Prostate..

[35] Godugu D., Chilamakuri R., Agarwal S. (2025). STAT3 axis in cancer and cancer stem cells: from oncogenesis to targeted therapies. Biochim. Biophys. Acta, Rev. Cancer..

[36] Xue X., Wang M., Cui J., Yang M., Ma L., Kang R., Tang D., Wang J. (2025). Glutathione metabolism in ferroptosis and cancer therapy. Cancer Lett..

[37] Alborzinia H., Flórez A.F., Kreth S., Brückner L.M., Yildiz U., Gartlgruber M., Odoni D.I., Poschet G., Garbowicz K., Shao C., Klein C., Meier J., Zeisberger P., Nadler-Holly M., Ziehm M., Paul F., Burhenne J., Bell E., Shaikhkarami M., Würth R., Stainczyk S.A., Wecht E.M., Kreth J., Büttner M., Ishaque N., Schlesner M., Nicke B., Stresemann C., Llamazares-Prada M., Reiling J.H., Fischer M., Amit I., Selbach M., Herrmann C., Wölfl S., Henrich K.O., Höfer T., Trumpp A., Westermann F. (2022). MYCN mediates cysteine addiction and sensitizes neuroblastoma to ferroptosis. Nat. Cancer..

[38] Zhao J., Ma Y.B., Xia R.H., Jiang Z.C., Zhou Y., Wang Y.N., Yang M.Y., Dai J.J., Zhu T., Pan L.B., Yuan L. (2025). Skullcapflavone II inhibits SLC1A4-Mediated L-Serine uptake and promotes mitochondrial damage in gastric cancer. Advanced science (Weinheim, Baden-Wurttemberg, Germany).

[39] Mukhopadhyay S., Biancur D.E., Parker S.J., Yamamoto K., Banh R.S., Paulo J.A., Mancias J.D., Kimmelman A.C. (2021). Proceedings of the National Academy of Sciences of the United States of America.

[40] Feng Z., Meng F., Huo F., Zhu Y., Qin Y., Gui Y., Zhang H., Lin P., He Q., Li Y., Geng J., Wu J. (2024). Inhibition of ferroptosis rescues M2 macrophages and alleviates arthritis by suppressing the HMGB1/TLR4/STAT3 axis in M1 macrophages. Redox Biol..

[41] Ramos S., Hartenian E., Santos J.C., Walch P., Broz P. (2024). NINJ1 induces plasma membrane rupture and release of damage-associated molecular pattern molecules during ferroptosis. EMBO J..

[42] Cao L., Tian H., Fang M., Xu Z., Tang D., Chen J., Yin J., Xiao H., Shang K., Han H., Li X. (2022). Activating cGAS-STING pathway with ROS-responsive nanoparticles delivering a hybrid prodrug for enhanced chemo-immunotherapy. Biomaterials..

[43] Shah R., Ibis B., Kashyap M., Boussiotis V.A. (2024). The role of ROS in tumor infiltrating immune cells and cancer immunotherapy. Metab. Clin. Exp..

[44] Jo Y., Shim J.A., Jeong J.W., Kim H., Lee S.M., Jeong J., Kim S., Im S.K., Choi D., Lee B.H., Kim Y.H., Kim C.D., Kim C.H., Hong C. (2024). Targeting ROS-sensing Nrf2 potentiates anti-tumor immunity of intratumoral CD8(+) T and CAR-T cells. Mol. Ther. :J. American Society of Gene Therapy..

[45] Zhou Y.C., Zhu H.L., Pang X.Z., He Y., Shen Y., Ma D.Y. (2025). The IL-6/STAT3 signaling pathway is involved in radiotherapy-mediated upregulation of PD-L1 in esophageal cancer. Ann. Clin. Lab. Sci..

[46] Wang C., Ju C., Du D., Zhu P., Yin J., Jia J., Wang X., Xu X., Zhao L., Wan J., Sun T., Yang L., Li H., He F., Zhou M., He J. (2025). CircNF1 modulates the progression and immune evasion of esophageal squamous cell carcinoma through dual regulation of PD-L1. Cell. Mol. Biol. Lett..

[47] (2021). Erratum: Metformin downregulates PD-L1 expression in esophageal squamous cell carcinoma by inhibiting IL-6 signaling pathway. Front. Oncol..

[48] Liu X., Tang R., Xu J., Tan Z., Liang C., Meng Q., Lei Y., Hua J., Zhang Y., Liu J., Zhang B., Wang W., Yu X., Shi S. (2023). CRIP1 fosters MDSC trafficking and resets tumour microenvironment via facilitating NF-κB/p65 nuclear translocation in pancreatic ductal adenocarcinoma. Gut.

[49] Liu C., Lai H., Chen T. (2020). Boosting natural killer cell-based cancer immunotherapy with selenocystine/transforming growth factor-beta inhibitor-encapsulated nanoemulsion. ACS Nano.

[50] Li W., Zeng Q., Wang B., Lv C., He H., Yang X., Cheng B., Tao X. (2024). Oxidative stress promotes oral carcinogenesis via Thbs1-mediated M1-like tumor-associated macrophages polarization. Redox Biol..

