# Supplementary material for: DMNQ induces ferroptosis and augments the efficacy of anti-PD-L1 immunotherapy in gastric cancer via the STAT3/SLC1A4 axis to mediate cysteine metabolism reprogramming
Source: Redox Biol. 2026 Jan 26;90:104055. doi: 10.1016/j.redox.2026.104055 (PMC12870803; doi:10.1016/j.redox.2026.104055)
Supplement: Multimedia component 1 [file mmc1.docx]

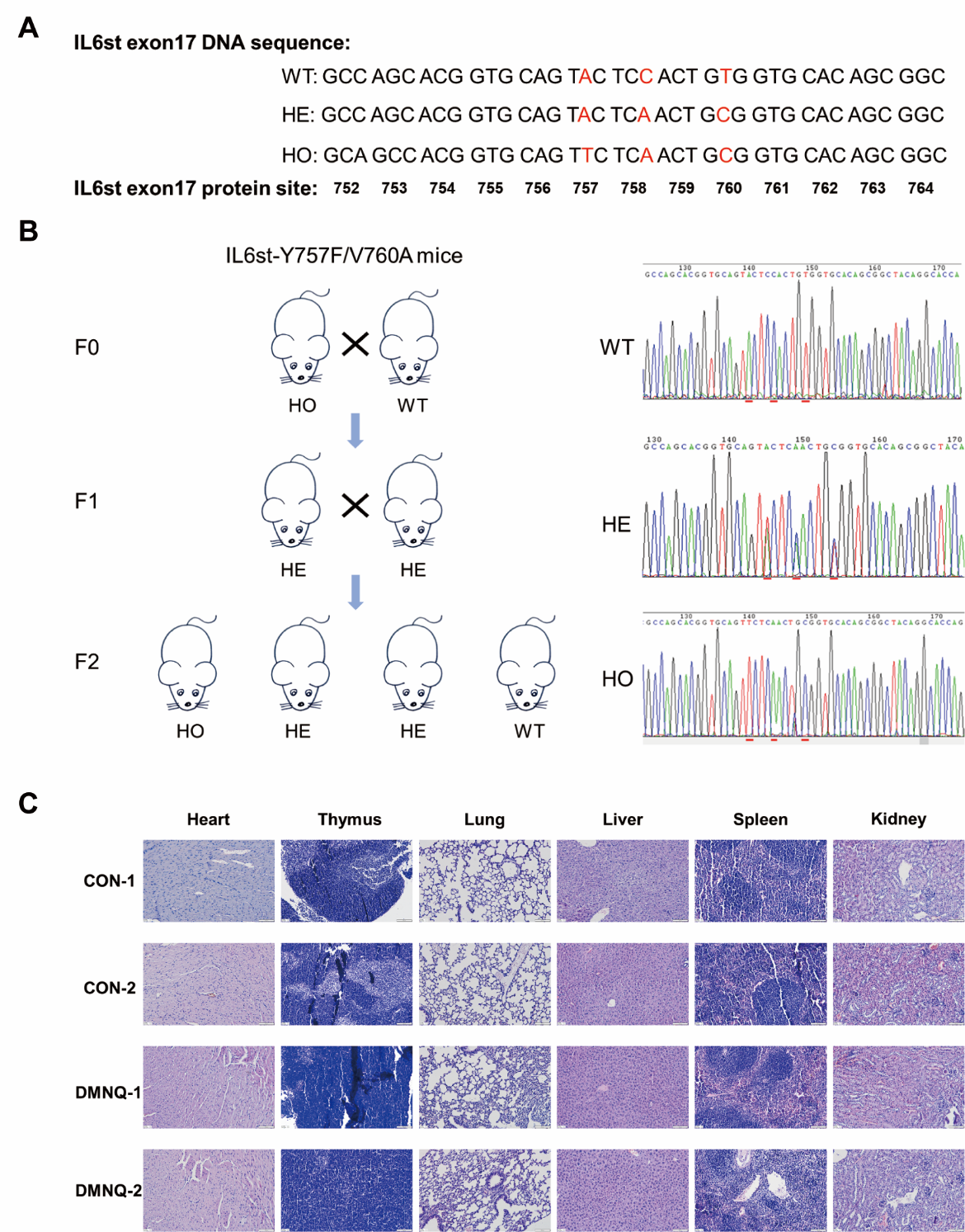
**Supplementary Information**

**Fig S1 Construction of transgenic C57BL/6J-IL6st ^em1(Y757F-V760A) Smoc mice^**

Mutation sites in C57BL/6J-IL6st^em1 (Y757F-V760A) Smoc^ mice: WT (wild type), HE (heterozygote), and HO (homozygote). **B** Breeding protocol for homozygous mice. **C** After DMNQ treatment, HE staining was performed on tissues (heart, thymus, lung, liver, spleen, and kidney) from transgenic mice to evaluate its biotoxicity.

**
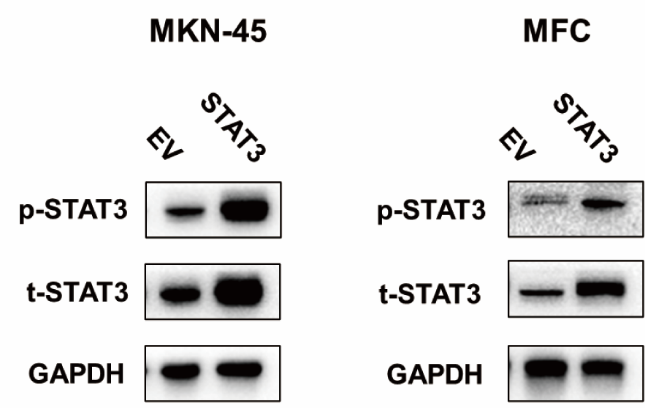
**

**Fig S2 Establishment of a STAT3-overexpressing gastric cancer cell line**

**Table S1. Target sequences of siRNA used in this study.**

| Gene symbol | Target sequences (5’→3’) |
| --- | --- |
| siSTAT3#1 (Forward) | GGAGCUGUUUAGAAACUUATT |
| siSTAT3#1 (Reverse) | UAAGUUUCUAAACAGCUCCTT |
| siSTAT3#2 (Forward) | GGUACAACAUGCUGACCAATT |
| siSTAT3#2 (Reverse) | UUGGUCAGCAUGUUGUACCTT |
| siSLC1A4 (Forward) | CGCGGUGUUCAUUGCGCAATT |
| siSLC1A4 (Reverse) | UUGCGCAAUGAACACCGCGTT |

**Table S2. ChIP-qPCR primers sequences.**

| Name | Primer sequences (5' → 3') |
| --- | --- |
| STAT3-SLC1A4 (Forward) | CGTTTCTTGCAGAAAAGAATGGT |
| STAT3-SLC1A4 (Reverse) | TCAGCTTTCACCCAGCAGAA |
